# The duration and breadth of antibody responses to 3-dose of inactivated COVID-19 vaccinations in healthy blood donors: An observational study

**DOI:** 10.3389/fimmu.2022.1027924

**Published:** 2022-11-01

**Authors:** Shanhai Ou, Zehong Huang, Miaoling Lan, Jianghui Ye, Jijin Chen, Huilin Guo, Jin Xiao, Shucheng Zhuang, Jiahuang Wu, Chuanlai Yang, Mujin Fang, Yingying Su, Ting Wu, Shengxiang Ge, Tong Cheng, Yongchang Zhang, Yongcai Lin, Yali Zhang, Guowei Chen, Quan Yuan

**Affiliations:** ^1^ Xiamen Blood Service, Xiamen, Fujian, China; ^2^ State Key Laboratory of Molecular Vaccinology and Molecular Diagnostics, School of Public Health, Xiamen University, Xiamen, Fujian, China; ^3^ National Institute of Diagnostics and Vaccine Development in Infectious Diseases, School of Life Science, Xiamen University, Xiamen, Fujian, China

**Keywords:** COVID-19, SARS-CoV-2, Omicron, COVID-19 vaccine, antibody duration, neutralization breadth

## Abstract

**Objectives:**

We aimed to evaluate the duration and breadth of antibodies elicited by inactivated COVID-19 vaccinations in healthy blood donors.

**Methods:**

We performed serological tests on 1,417 samples from 658 blood donors who received two (n=357), or three (n=301) doses of COVID-19 inactivated vaccine. We also accessed the change in antibody response before and after booster vaccination in 94 participants and their neutralization breadth to the current variants after the booster.

**Results:**

Following vaccination, for either the 2- or 3-dose, the neutralizing antibodies (nAbs) peaked with about 97% seropositivity approximately within one month but subsequently decreased over time. Of plasmas collected 6-8 months after the last immunization, the nAb seropositivities were 37% and 85% in populations with 2-dose and 3-dose vaccinations, respectively. The nAbs of plasma samples (collected between 2-6 weeks after the 3^rd^ dose) from triple-vaccinated donors (n=94) showed a geometric mean titer of 145.3 (95% CI: 117.2 to 180.1) against the ancestral B.1, slightly reduced by 1.7-fold against Delta variant, but markedly decreased by 4-6 fold in neutralizing Omicron variants, including the sub-lineages of BA.1 (5.6-fold), BA.1.1 (6.0-fold), BA.2 (4.2-fold), B.2.12.1 (6.2-fold) and BA.4/5 (6.5-fold).

**Conclusion:**

These findings suggested that the 3^rd^ dose of inactivated COVID-19 vaccine prolongs the antibody duration in healthy populations, but the elicited-nAbs are less efficient in neutralizing circulating Omicron variants.

## Introduction

Since being discovered in late 2019, SARS-CoV-2 has posed an enormous health burden globally. As of August 2022, more than 500 million COVID-19 confirmed cases and more than 6 million deaths (1). In addition to its inherent high transmissibility, the rapid mutagenic ability of SARS-CoV-2 makes controlling pandemics extremely difficult. To date, five variants, including Alpha, Beta, Gamma, Delta, and Omicron, have been classified as variants of concern (VOC) by World Health Organization due to the increased infectivity, virulence, or immune escape capacity (2). Currently, the worldwide predominantly circulating SARS-CoV-2 strain is the Omicron, which comprises several sub-lineages, including the BA.1, BA.2, and recently documented BA.2.12.1 and BA.4/5. Previous studies had demonstrated that Omicron variants largely evade immunity elicited by past infections of the ancestral virus or variants of other lineages (3, 4). Vaccination is promoted worldwide as the primary means of preventing COVID-19. Five COVID-19 vaccines have been approved in China due to the good safety and efficacy demonstrated in clinical trials, with two inactivated vaccines of CoronaVac (Sinovac) and COVILO (Sinopharm) gaining widespread use in China and several other countries (5). Until now, more than 88% of people in China completed the full 2-dose immunization, and over 700 million people have received the 3^rd^ dose (6).

As a major part of adaptive immunity, the antibody response is essential in preventing viral infections and disease development (7). For COVID-19 vaccines administered through the intramuscular route, although T-cell mediated immunity may also contribute to vaccine-elicited protection, positive correlations between the antibody titers (against viral spike) and vaccine efficacies have been evidenced in clinical trials (8–10). Therefore, antibody duration after vaccination could serve as a surrogate marker for vaccine efficacy persistence and clarify the need for booster vaccination. However, the long-term kinetics of antibody response to 2- or 3-dose inactivated vaccines are still largely unknown. Moreover, the profiles of antibodies elicited by inactivated COVID-19 vaccinations in neutralizing Omicron sub-lineages, particularly for newly emerged BA.2.12.1 and BA.4/5, remain to be explored. Aiming to deeply evaluate the duration and breadth of humoral immunity elicited by inactivated COVID-19 vaccines in healthy adults, we characterized the antibodies profiles in blood donors who received 2- or 3-dose vaccination in this study. The antibody levels of RBD-specific IgG (RBD-IgG), pan-immunoglobulin (pan-Ig), and neutralizing antibodies (nAbs) elicited by vaccinations were quantitatively measured. In addition, we also evaluated the cross-neutralization activities of plasmas, collected from 94 donors during 14-42 days (peaks) after the booster (third) dose, against the ancestral B.1, Delta, and various Omicron sub-lineages of BA.1, BA.1.1, BA.2, BA.2.12.1, and BA.4/5.

## Materials and methods

### Participants and samples

Serological tests and analyses were performed on 1,417 plasmas from 658 donors who donated blood in Xiamen. All involved donors received 2- or 3-dose of inactivated COVID-19 vaccines and met the blood donor health criteria of the National Health Commission of the People’s Republic of China (11). Their blood samples were tested negative in laboratory tests for HBV (HBsAg and HBV-DNA), HIV (HIV-Ag/Ab and HIV-RNA), HCV (HCV-Ab and HCV RNA), Treponema pallidum (TP-Ab), and HTLV (HTLV-1/2-Ab). Moreover, all involved donors were negative in the SARS-CoV-2 RNA tests and excluded for a history of past SARS-CoV-2 infection. This study was approved by the Ethics Committees of Xiamen Blood Service. Written informed consent was obtained from all participants.

### Pseudovirus-based neutralizing antibody (nAb) assays

Lentiviral-based pseudotyping particle (LVpp) bearing SARS-CoV-2 spikes were produced as previously described (12). Plasmids containing SARS-CoV-2 spike variant-expressing cassettes were generated by site-directed site-specific mutagenesis on a previously described vector (EIRBsMie-dSwtG, containing codon-optimized spike gene from MN908947.3 with D614G substitution). The 18aa from the C-terminus of the spike was replaced with a HiBit bioluminescent tag (14aa, GSGVSGWRLFKKIS) (13). Mutated spike-expressing cassettes, including that of Delta/B.1.617.2 (referring EPI_ISL_2723562), BA.1 (referring EPI_ISL_8324808), BA.1.1 (referring EPI_ISL_9640036), BA.2 (referring EPI_ISL_8253179), BA.2.12.1 (referring EPI_ISL_11704386) and BA.4/5 (referring EPI_ISL_11542465), were generated by introductions of the corresponding amino-acid substitutions of each strain into the B.1 backbone of EIRBsMie-dSwtG. Pseudoviruses of SARS-CoV-2 spike variants were produced in 293T-F17 cells by co-transfecting the spike-expressing plasmids, the packing plasmid of psPAX2, and the mNeonGreen reporter vector (pLVEF1αmNG) using Lipofectamine^®^ 3000 (Thermo Scientific). The supernatants were collected at 48 or 72 hours after transfection, filtrated by a 0.45-μm pore size filter, and were subsequently subjected to determine the titers in infecting huACE2-H1299 cells. Aliquot viral stocks were stored at -80°C freezer until use.

For neutralization antibody tests (NAT), plasma samples were heat-inactivated at 56°C for 30min before detection. Subsequently, serially-diluted samples (3-fold series dilutions, from 1:10 to 1:21,870) were pre-incubated with the pseudovirus inoculum (2,500 GFU/well) for 1 hour. The mixtures were further incubated with the huACE2-H1299 cells pre-seeded in 96-well cell culture plates at 37°C in a CO^2^ incubator. After a 2-day culture, cellular fluorescent images were acquired by using Opera Phenix high-content imaging system (PerkinElmer). For each well, the total (H2B-mRuby3-activated) and LVpp-infected (mNeonGreen-activated) cell numbers were determined by the Columbus Image analysis system (PerkinElmer). After normalization with the total cell number, the infection inhibition ratio of each sample at different dilutions was calculated by comparing it with the LVpp-only control. The nAb titer was defined as the maximum dilution fold required to achieve infection inhibition by 50% (ID_50_), which was determined by the 4-parameter logistic (4PL) regression using GraphPad Prism (version 9.3.1). An ID_50_≥20 was defined as the cutoff value to determine SARS-CoV-2 nAb seropositivity. And an ID_50_<5 was assigned a value of 5. The abovementioned NAT assay showed excellent performance with sensitivity and specificity of near 100% in our previous studies using pre-pandemic human samples and COVID-19-convalescent samples.

### Assays for RBD-specific pan-immunoglobulin (pan-Ig) and IgG antibodies

SARS-CoV-2 RBD-specific pan-Ig (RBD-pan-Ig) and IgG (RBD-IgG) antibodies were measured using commercial kits from Wantai BioPharm by magnetic particles-chemiluminescence immunoassay on Wan200+ fully automated system (Wantai, China), following the manufacturer’s protocols. The titers of RBD-pan-Ig and RBD-IgG were expressed as the cutoff index (COI).

### Statistical analysis

For the comparison of baseline characteristics, the Chi-Square statistic was used for categorical variables, and continuous variables were compared by using a two-sided Mann-Whitney *U*-test. The Kinetics of nAb, RBD-IgG, and RBD-pan-Ig were calculated by the GAM (generalized additive model) curve-fitting polynomial regression. Mann-Whitney *U*-test was used to compare the various antibody titer between two groups. The Friedman test with Dunn’s correction was applied to analyze differences among groups. The Spearman rank correlation coefficient was used for linear correlation analysis between the various antibody titer. Statistical analyses were conducted by GraphPad Prism (version 9.3.1) or R software (version 4.1.2).

## Result

### Study groups and baseline characteristics of the cohort

**Figure 1:**
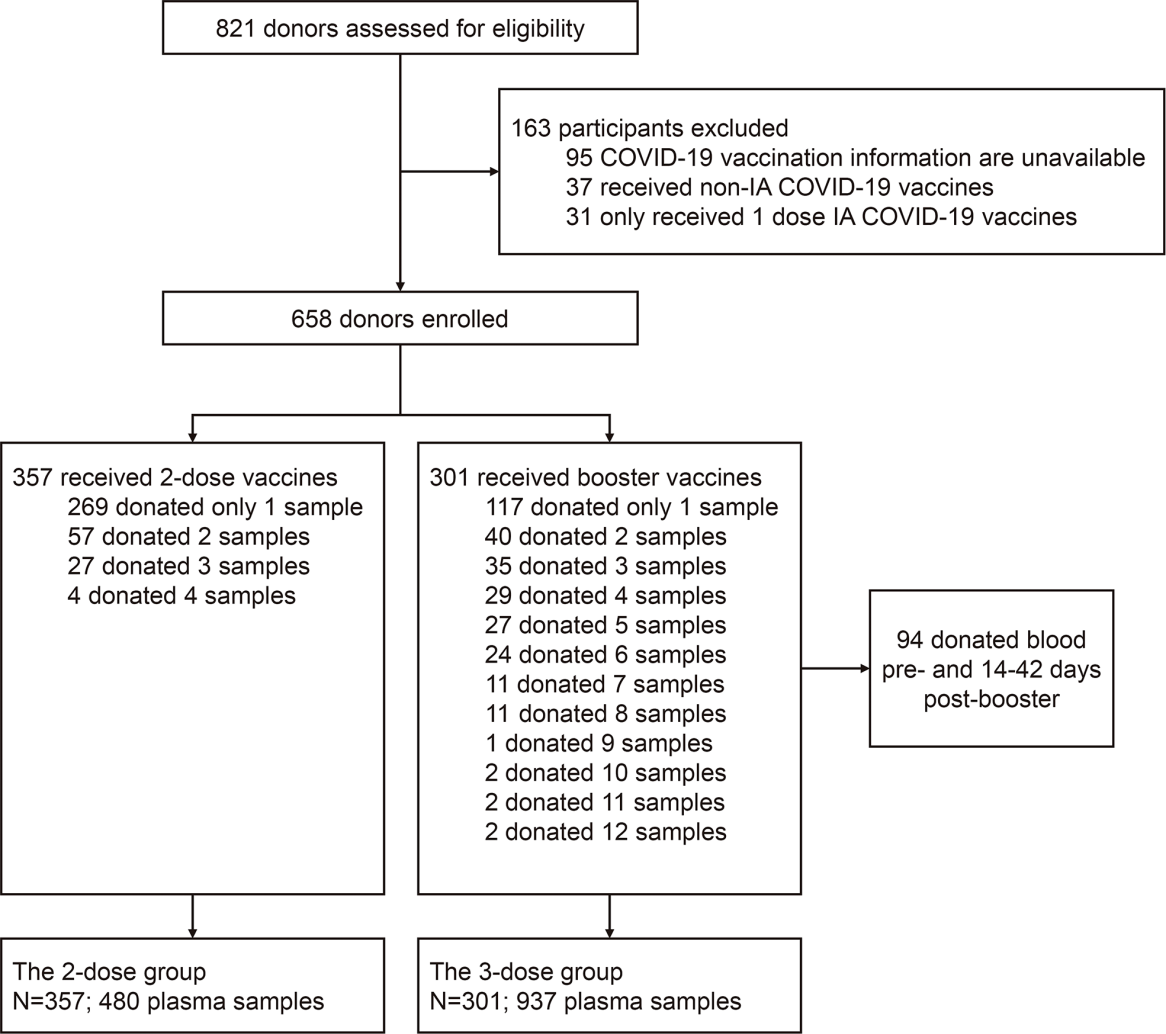
Flow chart describing the study cohort enrollment. The enrolled 658 donors were divided into two groups (the 2-dose and 3-dose groups) according to whether the people received the 3^rd^ booster vaccination during the study period. Among all subjects, 386 only donated their blood once, and the remaining 272 donated blood twice or more. IA-COVID-19 vaccine, inactivated COVID-19 vaccine.

In the study, 658 blood donors who received inactivated COVID-19 vaccine were enrolled, as the flowchart shown in **Figure 1**, with 357 participants receiving two doses of the vaccine (2-dose group) and 301 participants completing the booster (third) dose (3-dose group). The detailed baseline characteristics of the cohort are presented in **Table 1**. As the legal requirement for the age of blood donors is from 18 to 60 years old, the overall cohort had a median age of 38 years old (range: 19-59). The 3-dose group (41 years old) has a slightly higher median age than the 2-dose group (37 years old, **Table 1**). Approximately 80% of involved donors in the 2-dose or the 3-dose groups were males. The vast majority of participants were vaccinated using standard immunization procedures with a median interval of 28 days between the first and second doses. Most triple-vaccinated participants received the booster immunization about six months (median=195 days) after the second dose. Notably, 272 participants, including 88 in the 2-dose group and 184 in the 3-dose group, donated their blood more than once (**Figure 1**). Therefore, we included 1,417 plasma samples from these donors for analysis.

**Table 1:** Characteristics of the participants.

Characteristics	All donors (N=658)	2-dose (N=357)	3-dose (N=301)	P values^#^(2- v.s. 3-dose)
**Median age (range)**	38 (19-59)	37 (19-59)	41 (19-58)	0.0053
**Age groups (n, %)**				
18-30 years	140 (21.3)	85 (23.8)	55 (18.3)	0.0183
31-45 years	331 (50.3)	186 (52.1)	145 (48.2)	
46-59 years	187 (28.4)	86 (24.1)	101 (33.6)	
**Male (n, %)**	529 (80.4)	279 (78.2)	250 (83.1)	0.1388
**Blood type (n, %)**				
A	197 (29.9)	111 (31.1)	86 (28.6)	0.5813
B	179 (27.2)	98 (27.5)	81 (26.9)	
AB	54 (8.2)	32 (9)	22 (7.3)	
O	228 (34.7)	116 (32.5)	112 (37.2)	
**Interval between 1^st^ and 2^nd^ doses, days (median, IQR)**	28 (23-33)	27 (23-32)	28 (26-36)	<0.0001
**Interval between 2^nd^ and 3^rd^ doses, days (median, IQR)**	195 (188-233)	n/a	195 (188-233)	n/a

#The Chi-Square statistic was used for categorical variables. Continuous variables were compared by using a two-sided Mann-Whitney U-test. IQR, interquartile range; n/a, not applicable.

### Dynamic changes in the antibody levels elicited by 2- or 3-dose inactivated COVID-19 vaccines

**Figure 2:**
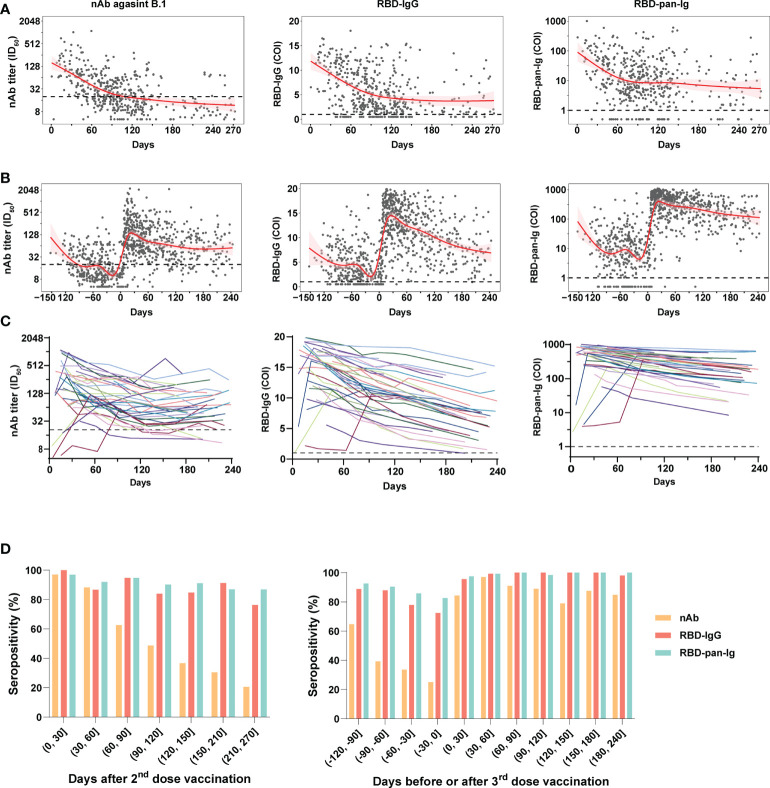
Dynamic changes of antibody response in blood donors with two- or three-dose inactivated COVID-19 vaccines. **(A, B)** The kinetics of the nAb (left panel), RBD-IgG (middle panel), and RBD-pan-Ig (right panel) titers in the 2-dose group **(A)** and 3-dose group **(B)** following the time since the last immunization. The 95% confidence intervals of the fitting curves are plotted as red-shaded areas. The broken blue lines represent the cutoff values. **(C)** Longitudinal changes of antibody titers in 37 individuals of the 3-dose group who donated their blood ≥4 times after the 3^rd^ dose immunization. Each line represents a connected line of antibody levels measured at different time points of the same donor. Dark shadows indicate the limits of detections (LODs). **(D, E)** The seropositivities of three antibody markers (indicated by columns with different colors) in the study populations following days since the 2^nd^ **(D)** or the 3^rd^ **(E)** dose vaccination. COI, cutoff index; ID_50_, half-maximal inhibitory dilution.

The nAb (against B.1), RBD-IgG, and RBD-pan-Ig levels of plasmas of donors were presented in **Figure 2** following days since the last immunization. In all samples, positive correlations in the levels were noted between any two of the three antibody markers (**Figure S1**). For the 2-dose group, the population antibody levels of nAb, RBD-IgG, and RBD-pan-Ig (**Figure 2A**) reached their peaks within four weeks after the 2^nd^ dose administration and then fell into decreasing over time. After approximately 3-4 months, the antibodies for either nAb, RBD-IgG, or RBD-pan-Ig gradually decline to a low-level plateau. For donors in the 3-dose group, our data showed the dynamic antibody changes at a population level within about 13 months, encompassing about five months before the 3^rd^ dose of vaccination and eight months after the booster immunization (**Figure 2B**). Following the booster vaccination, all three antibody markers rapidly increased and peaked at about three weeks post-vaccination (**Figure 2B**). In contrast to the 2-dose group, the antibody declines in the 3-dose group appeared slower with extended half-lives in the nAb (t_1/2_: 31 days v.s. 35 days), RBD-IgG (t_1/2_: 74 days v.s. 188 days), and RBD-pan-Ig (t_1/2_: 19 days v.s. 101 days) (**Table S2**). We further performed analyses using longitudinal samples from 37 individuals in the 3-dose group who donated their blood ≥4 times after the 3^rd^ dose immunization. The longitudinal antibody changes of these donors showed similar trajectory dynamics to that observed in pooled samples of the 3-dose group (**Figure 2C**).

Compared to the nAb, the RBD-IgG and RBD-pan-Ig were more stable over time, particularly when analyzing the seropositivity rate (**Figures 2D**). For both the 2-dose and 3-dose groups, the nAb seropositivities could reach about 97% at the peak phase. However, in the 2-dose group, the nAb seropositivity displayed sustained declines over time and only maintained 48.7% and 20.5% at 4 and 9 months after the last vaccination, respectively (**Figure 2D**). By contrast, at the same time points, the RBD-IgG was still detectable in 83.9% and 76.3% of samples, whereas the seropositivities of RBD-pan-Ig were 90.1% and 96.8%. Consistent with findings based on quantitative analyses, the 3^rd^ dose booster prolonged the seropositivity persistence of all three antibody markers. Among plasmas collected 6-8 months after the last dose immunization, the nAb seropositive in the 3-dose group was 85%, significantly higher than that observed in the 2-dose group (37%, p<0.001). In addition, the seropositivities of RBD-IgG and RBD-pan-Ig maintained nearly 100% by 6-8 months after the 3^rd^ dose immunization (**Figure 2D**). Overall, these data suggested the nAbs elicited by inactivated COVID-19 vaccines were less durable than RBD-specific binding antibodies. More importantly, the 3^rd^ dose of vaccination could improve the nAb duration in healthy populations.

### Neutralization breadth of antibodies elicited by 3-dose of inactivated COVID-19 vaccines

**Figure 3:**
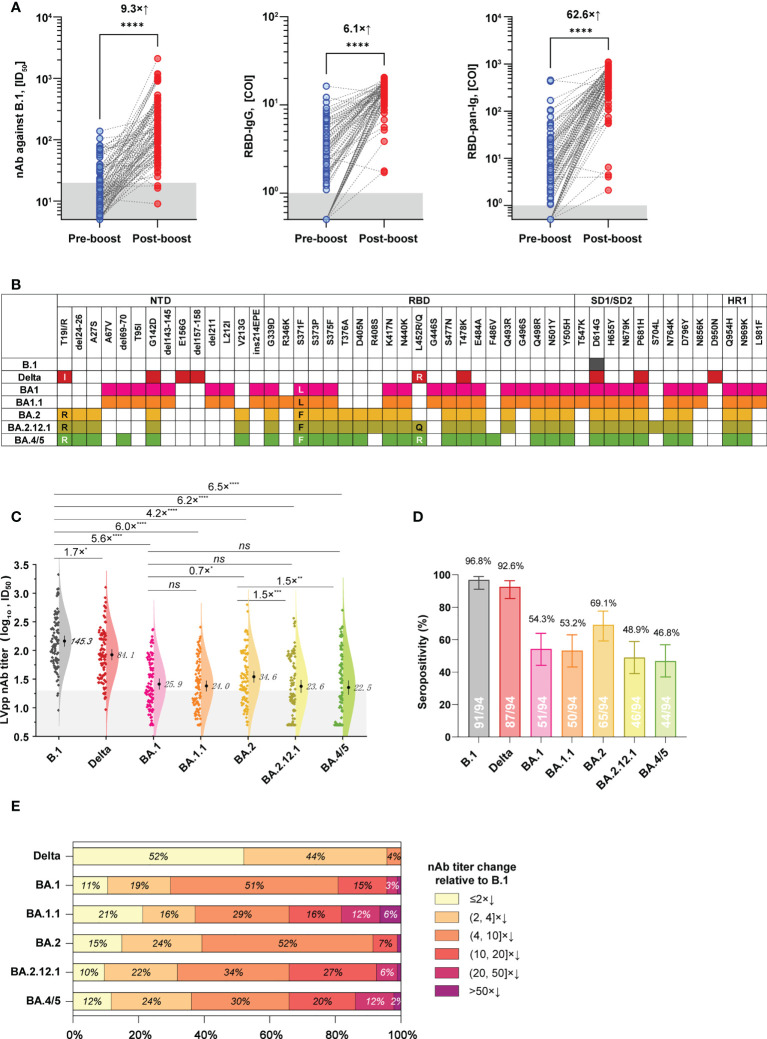
Cross-neutralization profiles of plasma samples from a subgroup in the 3-dose group against various spike variants. **(A)** The levels of nAb (against B.1, left panel), RBD-IgG (middle panel), and RBD-pan-Ig (right panel) in paired samples of the same donor were collected before and after the booster immunization. Dark shadows indicate the LODs. **(B)** Schematics of mutations presented in spikes of the B.1, Delta variant, and different Omicron sub-lineages. Colorized frames indicate the mutation presence at the corresponding amino-acid position. **(C)** Comparisons of the neutralizing activities of plasmas against the ancestral B.1 and different variants. The numbers show the nAb GMT against various viruses. The dark shadow indicates the LOD. **(D)** Seropositive percentages of these plasmas in neutralizing pseudoviruses bearing different spike variants. **(E)** Proportions of plasmas according to the strata of nAb decrease relative to B.1. COI, cutoff index; ID_50_, half-maximal inhibitory dilution; NTD, N-terminal domain; RBD, receptor binding site; SD1/SD2, subdomain 1/2; HR1, heptad repeat 1; *p<0.05; **p<0.01; ***p<0.001; ****p< 0.0001; ns, not significant (p>0.05). The combination of an “x” with an up arrow represents the folds of the relative increase in antibody, while the combination of an “x” with a down arrow indicates the opposite meaning, the folds of the relative decrease in antibody.

Among donors in the 3-dose group, 94 people donated their blood during pre- and post-booster (28 ± 14 days) administration. Based on these individuals and their samples, we further established a subgroup to analyze the cross-neutralization activities of 3-dose vaccine-elicited antibodies against SARS-CoV-2 variants. The baseline characteristics of this subgroup are presented in **Table S1**. After the booster vaccinations, plasmas from this subgroup presented geometric mean antibody titers (GMTs) of 145.3 (ID_50_, 95% CI: 117.2 to 180.1) for nAb against B.1, 13.4 (COI, 95% CI: 12.3 to 14.6) for RBD-IgG, and 356.8 (COI, 95% CI: 281.2 to 452.7) for RBD-pan-Ig, respectively. In contrast to the pre-booster antibody level of the corresponding donors, the booster vaccinations averagely induced a 9.3×, 6.1×, and 62.6× increase in the nAb, RBD-IgG, and RBD-pan-Ig, respectively (p<0.0001 for each comparison, **Figure 3A**).

We next tested the cross-neutralization activities of post-booster plasmas of this subgroup against the Delta variant and various Omicron sub-lineages, including BA.1, BA.1.1, BA.2, BA.2.12.1, and BA.4/5. Spike mutations of these variants are illustrated in **Figure 3B**. In contrast to the ancestral B.1, the Delta variant only showed weak immune evasion with a 1.7× nAb GMT decrease, but the Omicron variants of BA.1, BA.1.1, BA.2, BA.2.12.1, and BA.4/5 markedly attenuated the nAb GMT by 5.6×, 6.0×, 4.2×, 6.2×, and 6.5×, respectively (**Figure 3C**). On the other hand, the nAb seropositivities of these samples were 96.8%, 92.6%, 54.3%, 53.2%, 69.1%, 48.9%, and 46.8% against the B.1, Delta, BA.1, BA.1.1, BA.2, BA.2.12.1, and BA.4/5, respectively (**Figure 3D**). Notably, there was significant difference (p<0.05) for the nAb titers in neutralizing between BA.2 and BA.2.12.1 and BA.4/5 currently circulating, with about 1.5-fold decrease of GMT (**Figure 3C**). However, the proportions of over 10-fold nAb decrease relative to B.1 were 19.2% for BA.1, 34.0% for BA.1.1, 8.5% for BA.2, 34.0% for BA.2.12.1, and 34.0% for BA.4/5, respectively (**Figure 3E**).

## Discussion

Elucidating the kinetics of antibodies after vaccination has profound implications for the understanding of vaccine-induced immune responses and the adjustment of immunization strategies. For people who received inactivated COVID-19 vaccines, some studies had explored the kinetics of spike-specific binding antibodies up to about 6-month follow-up after 2-dose vaccinations (14–16). However, the long-term duration of protective immune response after 3-dose vaccinations is still unclear. This study utilized samples from blood donors to investigate antibody kinetics generated by inactivated COVID-19 vaccines. Among 2-dose vaccinated people, our data showed that the nAb half-life at the population level was about 30 days, and more than 60% of individuals turned negative after 4-5 months, suggesting that a booster vaccination at 3-6 months after the second dose is reasonable and necessary. These data regarding nAb dynamics are consistent with other observations of the inactivated COVID-19 vaccine, which also suggested a rapid decline in effectiveness over time after two doses of vaccination (17). The rapid decline of neutralizing antibodies was not only observed in people with inactivated vaccines but also in those immunized with mRNA and adenoviral vector vaccines (18–20). Notably, compared with the nAb, the RBD-IgG and RBD-pan-Ig decreased relatively slower, which were was detectable in the majority (>75%) of donors at 7-9 months after the 2^nd^ dose vaccination (**Figure 2D**). Although the underlying mechanisms for this phenomenon were still unknown, consistent findings were also noted in previous studies (14, 15). Among people with the 3^rd^ dose vaccination, we found that the nAb half-life slightly extended to 35 days, and 85% of participants remained detectable nAb at 6-8 months after the booster. By contrast, the half-life extension resulting from the 3^rd^ dose immunization was significantly longer for both RBD-IgG and RBD-pan-Ig than for the nAb. Although the RBD is the predominant nAb target, it should be noted that neutralizing antibodies only account for a fraction of antibodies with spike-binding capabilities. Moreover, methodological differences between functional assays (for nAb) and immunoassays (for RBD-IgG and RBD-pan-Ig) in measurements of the two classes of antibodies may also lead to their dynamic kinetics appearance. Our results demonstrated the 3^rd^ dose booster could improve the persistence of antibody response against SARS-CoV-2 in healthy adults, implying prolonged longevity of immune protection. Notably, a recent study based on real-world data from Hong Kong, China, has demonstrated the 3-dose immunizations of inactivated-vaccine provided significantly improved protection against severe COVID-19 compared to the 2-dose regimen (21). Taken together, for the inactivated COVID-19 vaccine, transitioning from a 2-dose primary series with an optional booster to a 3-dose primary series seems imperative, and all eligible individuals should receive a third dose.

In addition to immune response duration, there are concerns about the cross-neutralizing activities of vaccination-elicited antibodies against SARS-CoV-2 variants, particularly for the Omicron-related variants with multiplex spike mutations. Several previous studies have documented the Omicron variants are highly resistant to neutralizing antibodies derived from immune stimulations of ancestral spike antigens. Therefore, it’s not surprising to note significant nAb decreases of vaccinated plasmas in neutralizing Omicron variants in our study (**Figures 3C–E**). Our data suggested the Omicron-related variants of BA.1, BA.2, BA.2.12.1, and BA.4/5 efficiently evade the nAbs raised by 3-dose inactivated COVID-19 vaccines at a relatively comparable level (~4-6× GMT decrease, **Figure 3C**). However, the newly emerged BA.2.12.1 (with additional L452Q mutation) and BA.4/5 (with additional L452R, F486V, and R493Q reversion) appeared to cause more noticeable resistance than BA.2 to nAbs elicited by 3-dose inactivated COVID-19 vaccines, as more samples exhibited undetectable nAbs to the formers. The L452 and F486 mutations in BA.2.12.1 and BA.4/5 may contribute to the extended immune evasion capabilities of the two variants (22).

It should be noted that there are some limitations to our study. First, due to the inherent characteristics of blood donors, the subjects were mainly healthy adult males, excluding minors and the elderly, which may lead to the inability of this study to fully reflect the antibody response of the total population after vaccination. Second, the small but significant differences in age and the interval between the first and second doses of vaccinees may lead to some degree of differences in the antibody kinetic characteristics of the two groups. Third, the inactivated COVID-19 vaccine contained multiple viral proteins, antibodies, and cellular immune responses against non-spike proteins, including nucleocapsid, membrane, and envelope proteins, which may also contribute to vaccine protection efficiency and should be evaluated in further studies.

In summary, these results provide a comprehensive understanding of antibody response following two or three doses of inactivated COVID-19 vaccine. Our findings suggested the duration of antibody response raised by inactivated COVID-19 vaccines could be prolonged by the 3^rd^ dose. However, the magnitude of vaccine-elicited nAbs against Omicron variants is markedly reduced, particularly for the new BA.2.12.1 and BA.4/5. Next-generation COVID-19 vaccines with broad-spectrum antigenic coverage and a more convenient immunization approach [such as intranasal vaccines (23)] are urgently required.

## Data availability statement

The raw data supporting the conclusions of this article will be made available by the authors, without undue reservation.

## Ethics statement

The studies involving human participants were reviewed and approved by Ethics Committees of Xiamen Blood Service. The participants provided their written informed consent to participate in this study.

## Author contributions

Conceptualization: SO, YLZ, and QY; Investigation: SO, ML, ZH, JY, JC, HG, JX, SZ, JW, CY, MF, YCZ, YL, and YLZ; Formal analysis: ZH, SO, YS, YLZ, and QY; Writing original draft: ZH and QY; Reviewing and editing: SO, YLZ, YS, TW, SG, GC, and QY; Project administration: YCZ, YLZ, GC, and QY. Funding acquisition: SO, YLZ, and QY. All authors critically reviewed the manuscript and approved the final version.

## Funding

This project was supported by the National Natural Science Foundation of China (81902057, U1905205, and 81871316), Xiamen University President Fund (20720190124), grants of the Science and Technology Major Project (special for COVID-19 prevention and control) of Fujian province (2021Y0103) and Xiamen city (3502Z2021YJ013) in China.

## Conflict of interest

The authors declare that the research was conducted in the absence of any commercial or financial relationships that could be construed as a potential conflict of interest.

## Publisher’s note

All claims expressed in this article are solely those of the authors and do not necessarily represent those of their affiliated organizations, or those of the publisher, the editors and the reviewers. Any product that may be evaluated in this article, or claim that may be made by its manufacturer, is not guaranteed or endorsed by the publisher.
