# Supplementary material for: The duration and breadth of antibody responses to 3-dose of inactivated COVID-19 vaccinations in healthy blood donors: An observational study
Source: Front Immunol. 2022 Nov 1;13:1027924. doi: 10.3389/fimmu.2022.1027924 (PMC9663651; doi:10.3389/fimmu.2022.1027924)
Supplement: Supplementary file 1 [file DataSheet_1.docx]

**Table S1. Characteristics of the 3-dose vaccinated subgroup for analyzing neutralization breadth.**

| **Characteristics** | | **N=94** |
| --- | --- | --- |
| **Median age (range)** | | 45 (19-58) |
| **Age groups (n, %)** | |  |
|  | 18-30 years | 11 (11.7) |
|  | 31-45 years | 41 (43.6) |
|  | 46-60 years | 42 (44.7) |
| **Male (n, %)** | | 78 (83.0) |
| **Blood type (n, %)** | |  |
|  | A | 28 (29.8) |
|  | B | 23 (24.5) |
|  | AB | 8 (8.5) |
|  | O | 35 (37.2) |
| **Interval between 1^st^ and 2^nd^ doses, days (median, IQR)** | | 28 (24-34) |
| **Interval between 2^nd^ and 3^rd^ doses, days (median, IQR)** | | 194 (188-212) |
| **Sampling time post booster, days (median, IQR)** | | 25 (17-29) |

IQR, interquartile range.

**Table S2. Population-level half-lives of antibody markers elicited by two of three doses of inactivated COVID vaccines.**

|  | | nAb against B.1 | RBD-pan-Ig | RBD-IgG |
| --- | --- | --- | --- | --- |
| 2-dose | T_1/2_ (days) | 31 | 19 | 74 |
| 3-dose | T_1/2_ (days) | 35 | 101 | 188 |


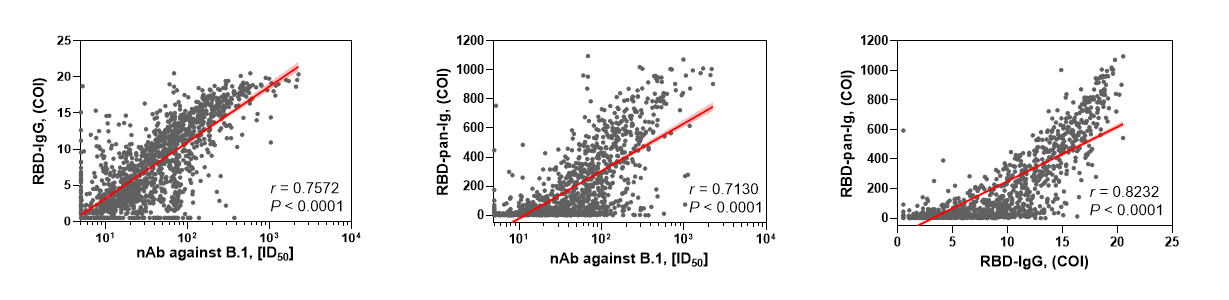


**Figure S1.** **Correlation analyses of the levels of three antibody markers among all involved samples. (A)** The nAb v.s. RBD-IgG. **(B)** The nAb v.s. RBD-pan-Ig. **(C)** RBD-IgG v.s. RBD-pan-Ig. COI, cutoff index; ID_50_, half-maximal inhibitory dilution. All 1,417 samples were measured using neutralization assays. Ten samples were not measured by RBD-IgG and RBD-pan-Ig tests due to the insufficient sample volume for loading on the automatical immunoassays. Therefore, data from 1,407 samples were included in the analyses.
